# Multi-trait Analysis of GWAS Expands Eosinophilic Esophagitis Genetic Susceptibility and Polygenic Risk Scores

**DOI:** 10.21203/rs.3.rs-6630283/v2

**Published:** 2025-07-18

**Authors:** Michael P. Trimarchi, Bahram Namjou-Khales, Netali Ben-Baruch Morgenstern, Mark Rochman, Xiaoting Chen, Garrett Osswald, John Besse, Molly Shook, Julie Caldwell, Michael Lape, Tetsuo Shoda, Matthew T. Weirauch, Melanie Ruffner, Gregory Constantine, Lisa J. Martin, Leah C. Kottyan, Marc E. Rothenberg, Consortium of Eosinophilic Gastrointestinal Disease Researchers

**Affiliations:** Cincinnati Children’s Hospital Medical Center; Cincinnati Children’s Hospital Medical Center; Cincinnati Children’s Hospital Medical Center; Cincinnati Children’s Hospital Medical Center; Cincinnati Children’s Hospital Medical Center; Cincinnati Children’s Hospital Medical Center; Cincinnati Children’s Hospital Medical Center; Cincinnati Children’s Hospital Medical Center; Cincinnati Children’s Hospital Medical Center; Cincinnati Children’s Hospital Medical Center; Cincinnati Children’s Hospital Medical Center; Cincinnati Children’s Hospital Medical Center; Children’s Hospital of Philadelphia; National Institute of Allergy and Infectious Diseases; Cincinnati Children’s Hospital Medical Center; Cincinnati Children’s Hospital Medical Center; Cincinnati Children’s Hospital Medical Center; CEGIR

**Keywords:** Eosinophilic Esophagitis, Allergy, Genome-wide Association Studies, Gene Expression Profiling

## Abstract

Atopic diseases, including eosinophilic esophagitis (EoE), are driven in part by genetic susceptibility. We performed a genome-wide association study (GWAS) of 1,757 EoE and 14,467 population controls, identifying 11 independent genetic risk variants spanning 8 EoE risk loci (p < 5×10^− 8^), including 3 new loci. A multi-trait analysis of GWAS (MTAG) of EoE and other atopic diseases including over 450,000 subjects from the UK Biobank study identified 33 independent EoE genetic risk variants spanning 24 loci, including 14 novel loci. Functional studies nominated 90 EoE candidate genes, some involved in unexpected pathoetiology beyond type 2 immunity. A polygenic risk score derived from the MTAG replicated high risk of EoE compared with PRS derived from GWAS alone (OR 11.57 [6.90–19.40] in the top vs. bottom decile). An interactive tool (EGIDExpress) was developed to enable dataset queries and visualization. These findings offer expanded insight into EoE genetic risk and pathoetiology, underscore the genetic interplay of EoE with common atopic diseases, and provide a public resource that will advance the allergy field.

## Introduction

Eosinophilic esophagitis (EoE) is a chronic, antigen-induced allergic inflammatory disorder affecting approximately 1 in 700 individuals and characterized by the infiltration of eosinophils in the esophagus ^1–6^. EoE is strongly linked to allergic inflammation, as evidenced by the success of elimination diets that remove food allergens, the frequent co-occurrence of EoE with other allergic conditions like asthma and atopic dermatitis, and animal models in which allergen exposure induces EoE ^5,7,8^. Furthermore, studies have shown the crucial role of type 2 immune mediators, such as interleukin 4 (IL-4) and interleukin 13 (IL-13), in both experimental and clinical settings ^9–12^. The inflammatory cascade associated with EoE leads to impairment of the esophageal epithelial barrier, characterized by dilated intercellular spaces, dysregulated gene expression, basal cell hyperplasia, local tissue eosinophilia, disrupted epithelial differentiation, and fibrostenosis, if left untreated ^13,14^.

The pathogenesis of EoE is primarily driven by food hypersensitivity and allergic responses and influenced by genetic and environmental factors ^15–17^. Genetic association studies have nominated a limited number of risk loci at genome-wide significance ^18–24^. Some risk loci are shared with other allergic diseases. For instance, variants at the 5q22 (*TSLP/WDR36*), 11q13 (*LRRC32/C11orf30*), and 12q13 (*STAT6*) loci are associated with asthma and atopic dermatitis and have been consistently associated with EoE risk ^19,24^. To date, the robustly associated 2p23 (*CAPN14*) EoE risk locus is unique to EoE and not implicated in other atopic diseases ^19,20,24,25^. Other EoE loci, such as 19q13 (*ANKRD27*) and 16p13 (*CLEC16A*), have been replicated within individual studies, though more research is needed to fully understand their contribution ^19,21^. Gene-gene interactions, particularly between *IL4* and *TSLP*, and gene-environment interactions have also been shown to have significant roles in EoE development ^26,27^.

To further investigate the genetic underpinnings of EoE, we conducted a genome-wide association study (GWAS) aimed at replicating and refining known risk loci and also identifying new ones. This study included between six hundred thousand and two million genotyped genetic variants in independent sets of cases and controls of European ancestry with a genome-wide coverage of five to eight million genetic variants after imputation across two cohorts including 1,757 individuals with EoE who were assessed in the context of population, atopic, and non-atopic controls. Furthermore, because EoE is genetically correlated with other allergic diseases ^22^, we performed a multi-trait analysis, examining loci shared between EoE and other atopic diseases in a larger cohort of more than 93,000 individuals with and 362,000 without history of atopic disease using the United Kingdom Biobank (UKB) dataset. Each analysis was bolstered by functional studies that integrated expression quantitative trait loci (eQTL) data and chromatin interaction models to predict causal genes linked to EoE risk loci. Accordingly, we identified 90 genes associated with EoE risk, including 47 not previously implicated in the disease. Furthermore, the multi-trait analysis identified a substantial polygenic risk for EoE; for example, those in the fifth decile had a 5-fold increased risk and those in the top decile had greater than an 11-fold increased risk of EoE compared to those in the bottom decile. These findings expand our understanding of the genetic architecture of EoE and highlight the utility of integrating genetic data across related atopic conditions. Additionally, the results of this study are available through an online interactive tool EGIDExpress. Taken together, our findings have uncovered a large set of genetic risk factors that contribute to the pathoetiology of EoE and provide a public resource for the field.

## Results

### EoE GWAS study

**Figure 1:**
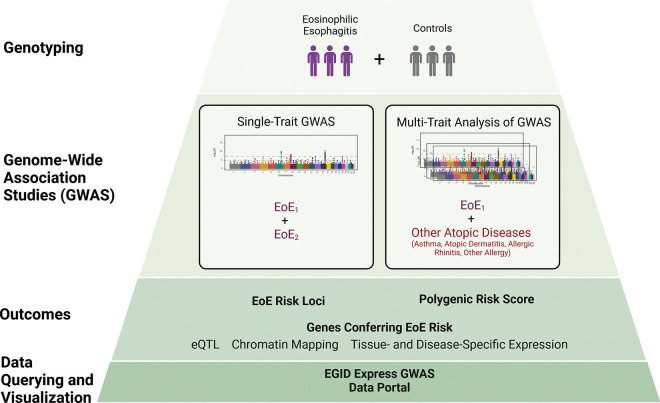
Study overview.

**Figure 2:**
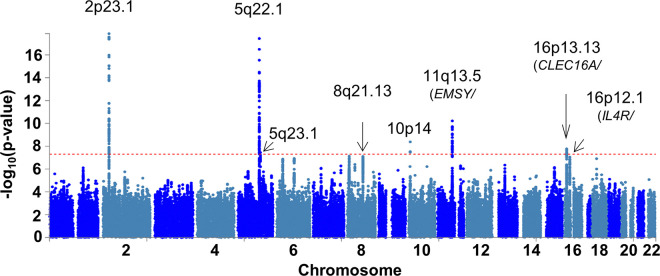
Risk loci identified in the EoE-GWAS. An EoE genome-wide association study (EoE_T_) was conducted comprising EoE (n = 1,757) and population controls (n = 14,467) genotyped and imputed genome-wide. The p values of each variant are plotted by chromosomal position. Dotted red line indicates genome-wide significance (p < 5e-8). Parentheses indicate representative mapped genes at each risk locus.

To identify new EoE genetic risk loci and replicate previously described loci, we assembled two independent EoE case-control cohorts (herein referred to as EoE_1_ and EoE_2_, as well as the merged cohort EoE_T_) encompassing a total of 1,757 participants with EoE and 14,467 population controls without EoE and performed a GWAS (Fig. 1, Figure S1, Figure S2, Table S1). Eleven lead variants across 8 EoE risk loci met genome-wide significance (p < 5×10^− 8^): 2p23.1, 5q22.1, 5q23.1, 8q21.13, 10p14, 11q13.5, 16p13.13, and 16p12.1 (Fig. 2, Table 1A). An additional 14 lead variants across 17 EoE risk loci showed putative association (10^− 6^ > p > 5×10^− 8^, Table 1B).

Two risk loci displayed multiple, independent associations (3 at 2p23.1, 2 at 11q13.5). At 2p23, conditional logistic regression analysis revealed multiple variants independently associated with EoE. Two variants that were independently associated with EoE (rs77569859, rs11124250) at 2p23 within the gene body of *CAPN14* were near or overlapping previously reported variants ^24^. A third association at rs77746069 between exon 1 and 2 of the downstream gene *GALNT14* demonstrated residual attenuated association after conditioning on the other two variants (Figure S3). At 11q13.5, we replicated two independent associations (rs61894547, rs7936323, linkage disequilibrium [LD] r^2^ < 0.1) that had been previously reported ^24^.

Three novel, genome-wide significant EoE risk loci were mapped to the following chromosomal bands: 8q21.13, 10p14, and 16p12.1. The 8q21.13 locus has previously been associated with atopic disease and eosinophil counts ^28,29^; chromatin interaction data suggest a long-range interaction with the gene *ZBTB10*, a transcription factor ^30^ (Table S2). Variants at 10p14 have been previously associated with EoE in the vicinity of *ITIH5* and *GATA3* ^24^; however, this study identified an independent association (LD r^2^ < 0.01) over 1 MB away in an intergenic region, a locus that has been associated with eosinophil counts and atopic disease ^28,29^. The 16p12.1 risk locus encompasses a 120-kb region spanning *KDM8*, *NSMCE1*, and *IL4R* that has been associated with asthma ^31,32^; the region contains eQTLs for both *NSMCE1* (expressed in esophageal mucosa, monocytes, CD4 + T cells) and *IL4R* (expressed in esophageal mucosa, monocytes), and associated variants also interact with *IL4R* via chromatin looping. Independent significant variants at this locus were independent of the Q576R amino acid substitution (rs1801275) associated with atopic dermatitis and asthma (LD r^2^ < 0.1) ^33,34^.

Five of the EoE risk loci identified have been previously reported: 2p23.1, 5q22.1, 5q23.1, 11q13.5, and 16p13.13. 2p23.1 includes *CAPN14*, which encodes for a protease that is uniquely expressed in the esophageal epithelia as a function of IL-13–driven inflammation, EoE disease activity, and EoE risk genotype ^19,20,35–37^. The 5q22.1 risk locus encompasses a 200-kb region of LD overlapping *TSLP*, *WDR36*, and *CAMK4*; this locus is also known to be associated with atopic disorders and peripheral blood eosinophil counts ^38,39^. TSLP is a pro-atopy alarmin ^40^ that has an important role in the development and activation of many immune cells and promotes Th2 cell development through IL-4 activation ^41^ and proliferation of pathogenic effector memory Th2 cells ^42^. The association at 5q23.1 ^24^ lies within a weakly characterized intergenic region. The risk locus at 11q13.5 is located between *EMSY* (also known as *C11orf30*) and *LRRC32*; functional studies suggest a role for both genes in EoE ^24,43^, and the locus is also associated with eosinophil counts ^28,44^, asthma ^45^, atopic dermatitis ^46^, allergic rhinitis ^27^, serum IgE levels ^47^, and Crohn disease ^48^. The 16p13.13 risk locus ^24^ overlaps *CLEC16A* and is associated with eosinophil counts and atopic disease; associated variants also include eQTLs with *DEXI* in CD4 + T cells and monocytes. Though CAPN14 likely contributes to the tissue specificity of EoE, the EoE risk genes *TSLP*, *EMSY*, *LRRC32*, and *CLEC16A* are hypothesized to have roles in numerous allergic diseases ^49^.

Cases with EoE were also compared to controls with and without atopic disease (Table S5). Four additional risk loci were identified using non-atopic controls −3q28, 5q31.1, 8p23.1, 15q23)–of which 5q31.1 has been previously reported (Table S2) ^22^. The 3q28, 5q31.1, and 8p23.1 risk loci were confirmed by a multi-trait analysis approach (see next section).

### Multi-trait analysis of EoE and other atopic disease GWAS (EoE-atopy-MTAG)

EoE is a relatively rare atopic disease commonly associated with other atopic comorbidities ^50,51^;86% of the participants with EoE in this study reported at least one other atopic condition (Table S1). A recent EoE GWAS by Chang *et al*. showed a genetic correlation (r_g_) of over 50% with other atopic diseases, including allergic asthma, allergic rhinitis, and atopic dermatitis ^22^. Consistent with previous findings, we found a strong genetic correlation in this study using an LD score regression approach (Table S3).

The multi-trait analysis of GWAS (MTAG) method was developed to augment the power of single-trait genetic studies by incorporating information from highly correlated traits (r_g_ > 0.5) ^52^. Unlike traditional meta-analysis, which generates a single consensus effect estimate per variant across all studies and often reduces power when comparing different traits, MTAG offers separate effect estimates for each trait and is robust to sample overlaps.

**Figure 3:**
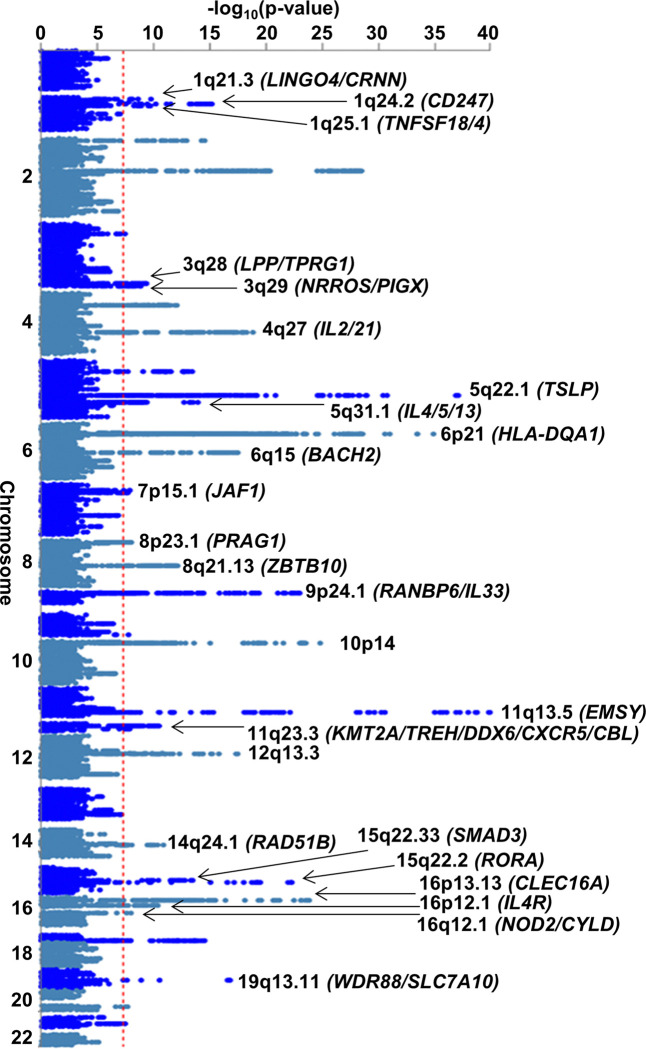
Risk loci identified in the EoE-GWAS. An EoE genome-wide association study (EoE_T_) was conducted comprising EoE (n = 1,757) and population controls (n = 14,467) genotyped and imputed genome-wide. The p values of each variant are plotted by chromosomal position. Dotted red line indicates genome-wide significance (p < 5e-8). Parentheses indicate representative mapped genes at each risk locus.

The EoE and other atopic disease MTAG (EoE-atopy-MTAG) presented here tested the hypothesis for each variant that EoE and atopic disease share a common genetic etiology. EoE_1_ (n = 1,033 participants with EoE) was compared to atopic association studies derived from the UKB study (n = 93,386 participants with atopic disease) (Table S5). Leveraging whole-genome imputation, over 6 million variants were assessed. Fifty-six independent variants met genome-wide significance (p < 5×10^− 8^), and 33 remained after removing 23 variants that did not meet a minimum threshold in the EoE_1_ cohort. These 33 lead variants spanned 24 genome-wide significant risk loci with robust evidence in EoE (p < 5×10^− 8^), EoE_1_ p < 0.05) (Fig. 3). Of the 8 risk loci identified in the analysis of EoE_T_ (EoE n = 1,757), 6 were significant in the multi-trait analysis, highlighting the strong genetic correlation across allergic diseases. Variants at 2p23.1 and 5q23.1 identified in the case-control study were not significant in EoE-atopy-MTAG, supporting tissue specificity. Of the 24 risk loci identified by the MTAG analysis, 13 were novel in EoE (1q21.3, 1q24.2, 1q25.1, 3q28, 3q29, 4q27, 7p15.1, 8p23.1, 9p24.1, 11q23.3, 14q24.1, 16q12.1, 19q13.11) (Table 2A), 2 were replicated in EoE_T_ (8q21.13, 10p14), and 9 were previously reported ^18–23^ (5q22.1, 5q31.1, 6q15, 11q13.5, 12q13.3, 15q22.2, 15q22.33, 16p13.13, 16p12.1) (Table 2B). Of the non-novel loci, 12q13.3 (*STAT6*; rs3024971) showed a new independent association (LD r^2^ < 0.1) 10 kb downstream of the variant reported by Sleiman *et al*. ^25^.

**Figure 4:**
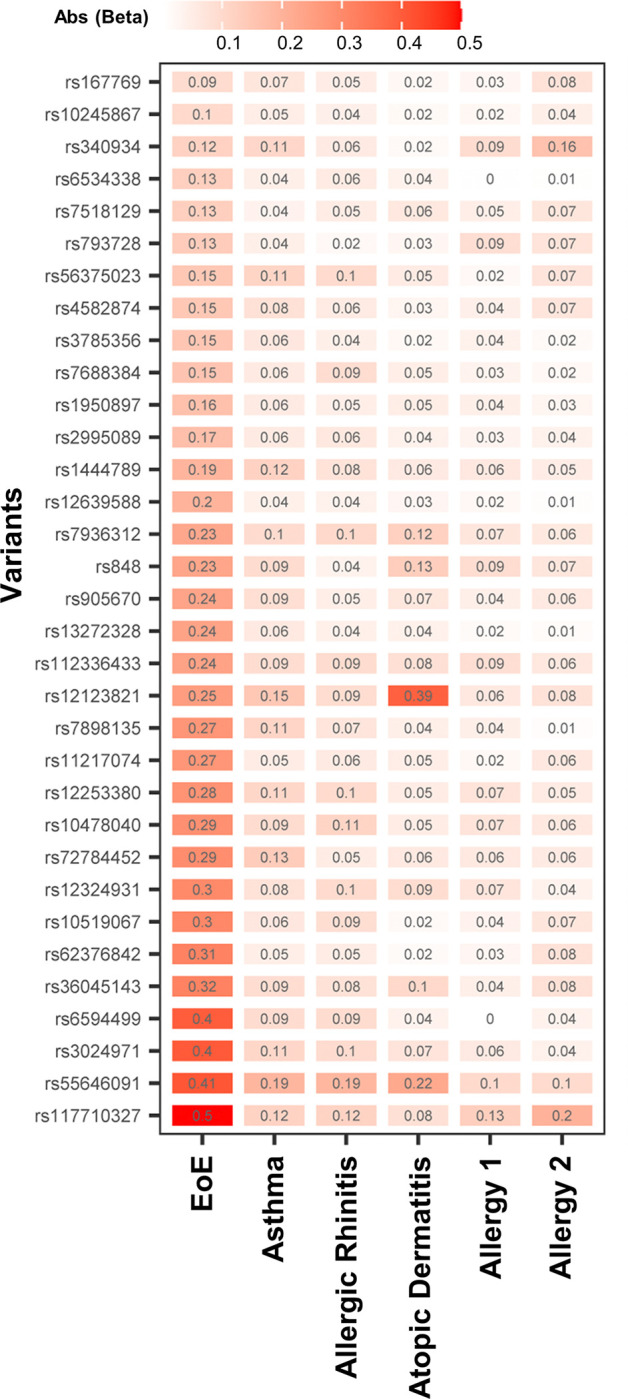
Effect sizes of EoE risk loci identified in the multi-trait analysis of GWAS. A multi-trait analysis was performed between EoE_1_ (EoE n = 1,033) and other allergic diseases (asthma, allergic rhinitis, atopic dermatitis, allergy-not-specified) using MTAG. Effect size for the 33 lead variants (MTAG p < 5e-8, EoE_1_ p < 0.05, LD r^2^ < 0.1) was plotted across each cohort in the EoE-atopy-MTAG analysis. The color intensity denotes effect size. Lead variant rs number identified in Y axis and effect size (Abs (Beta)) are shown in each rectangle. Abs: absolute value.

To determine whether the EoE association seen in the EoE-atopy-MTAG was driven primarily by other atopic diseases, we compared the normalized effect size for the 33 EoE-atopy-MTAG lead variants across each of the constituent cohorts (Fig. 4A). Effect size was largest in EoE for 32 of 33 lead variants that met study thresholds for significance. Median effect size was substantially larger in EoE than other atopic diseases (EoE beta 0.24 vs. 0.09 for asthma, the next largest; p < 0.001 Repeated Measures ANOVA on Ranks and p < 0.05 Tukey test) (Figure S4). In summary, the EoE risk loci identified by the MTAG analysis were not driven primarily by other atopic diseases.

The genes encoded at the 13 new EoE genetic risk loci suggest biological mechanisms that lead to the development and pathogenesis of EoE. Notably, the 1q21.3 risk locus maps to an expansive 600-kb block within the epidermal differentiation complex ^53^ including *FLG* and *FLG2*, which have known roles in esophageal barrier function in EoE ^54,55^. EoE risk genes were expressed in both epithelial and immune cells, with many of the genes showing supporting evidence in mechanistic studies and/or differential expression in patients with EoE compared to controls (Table S6).

Interactive zoom plots are available on the LocusZoom platform for exploration of the association of individual variants in a genomic context for the EoE_T_ and EoE-atopy-MTAG analyses.

### Sex-stratified association analysis of EoE risk loci

Due to the male predominance of EoE, we performed a sex-stratified analysis of EoE_T_ risk loci to test the hypothesis that some genetic risk would be amplified in males relative to females. A genome-wide, sex-stratified discovery analysis (Figure S5) identified one male-specific locus with genome-wide significance: 12q24.31 mapping to *SCARB1* (p = 2.01×10^− 10^); odds ratio [OR] = 1.44, 95% confidence interval [CI] 1.29–1.62, Figure S5A) with no association observed in females (p = 0.75, OR = 0.97, 95% CI 0.81–1.16). *SCARB1* encodes for a scavenger receptor that binds cholesterol esters and has been implicated in inflammatory responses ^56^. Because there were fewer females than males with EoE and a discovery analysis was likely underpowered to detect female-specific associations at genome-wide significance, a more limited sex-stratified analysis was performed for the genome-wide significant risk loci identified in EoE_T_, EoE-atopy-MTAG, and the *SCARB1* risk locus (Table S4). No other sex-specific associations were observed with overlapping 95% CIs. Together, these results indicate male-specific association at 12q24.31 and no evidence of loci with female-specific association.

### A validated polygenic risk score (PRS) for EoE

**Figure 5:**
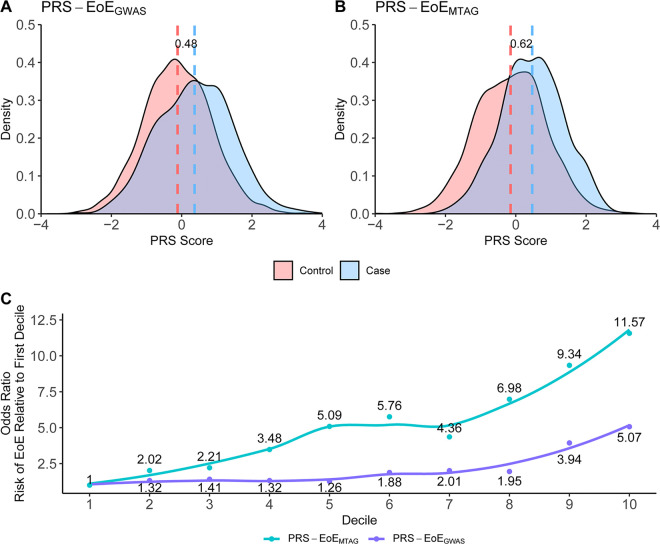
Comparison of polygenic risk scores derived from EoE alone vs. MTAG in an independent cohort. Polygenic risk scores (PRS) were constructed using a machine learning method on two association studies, EoE GWAS alone (EoE_1_) to create PRS-EoE_GWAS_ or the multi-trait analysis of EoE and atopic diseases (EoE-atopy-MTAG) to create PRS-EoE_MTAG_. These two PRS were both evaluated as shown in an independent dataset (EoE_2_) (EoE n = 724, control n = 3,113). **(A, B)** Density plots showing the distribution of PRS between EoE and controls. Dashed lines represent mean PRS for EoE (blue, case) and control (red), and the numerical label quantifies the difference. **(C)** Odds ratios vs. the first decile are shown for each PRS and represent the risk of EoE relative to the first decile. Curves represent smoothed local regression plotted using the LOESS (locally estimated scatterplot smoothing) function.

GWAS identifies population-level associations between specific genetic risk loci and disease, whereas polygenic risk score (PRS) can identify overall genetic risk burden for individuals. Two EoE PRS were developed using either case-control GWAS data from the EoE_1_ cohort (PRS-EoE_GWAS_, derived from case-control statistics in EoE_1_) or MTAG data from the EoE_1_ cohort (PRS-EoE_MTAG_, derived from the EoE-atopy-MTAG summary statistics in EoE_1_). Both the PRS-EoE_GWAS_ and PRS-EoE_MTAG_ were assessed in the independent EoE_2_ cohort. Globally, the EoE_2_ cohort had a significantly higher median standardized PRS-EoE_GWAS_ in cases than controls (68% vs. 45%, respectively, p < 0.0001) (Fig. 5A–B). A comparison of the two EoE PRS percentile distribution between cases and controls and the risk prediction per each decile in the EoE_2_ cohort are shown in Fig. 5. The discrimination between the top 10th percentile and the bottom 10th percentile of the PRS-EoE_MTAG_ (OR = 11.57, 95% CI 6.90–19.40) was superior to that of the PRS-EoE_GWAS_ (OR = 5.07, 95% CI 3.41–7.54) (Fig. 5C). The increased odds risk per standard deviation of the PRS-EoE_MTAG_ and PRS-EoE_GWAS_ was 1.71 (95% CI 1.52–1.92) and 1.65 (95% CI 1.51–1.80), respectively. Notably, the PRS-EoE_MTAG_ outperformed the PRS-EoE_GWAS_ in predicting EoE risk (p < 0.0001), further supporting the value of the multi-trait approach.

### Mapping EoE risk loci to candidate genes

Independent significant variants from the association studies presented above were mapped to genes using three methods: eQTL (esophageal mucosa and immune cells), chromatin interaction (esophagus and immune cells), and position (Table 3). A total of 90 candidate EoE risk genes were mapped, including 26 that were mapped from EoE_T_ (Tables S2 and S9). A presentation of each of the 90 candidate genes in the context of originating association study and mapping method is provided in Table S10 and Figure S6.

### Expression and function of EoE candidate risk genes in disease

**Figure 6:**
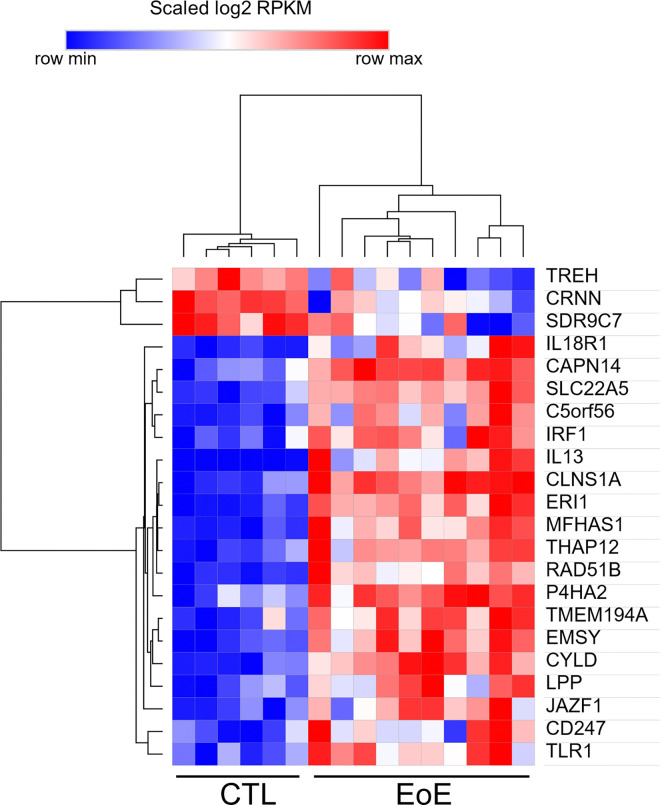
Expression of EoE risk candidates as a function of disease state. Disease-dependent expression is shown for 22 differentially expressed genes (DESeq2-adjusted p < 0.05, fold change >2). These genes were selected from 59 candidate genes (of 90 total that met the expression threshold of RPKM >1) in RNA sequencing of EoE (n = 10) and control (CTL, n = 6) biopsies. RPKM, reads per kilobase of transcript per million mapped reads.

To understand how the EoE risk loci might influence EoE biology, we first examined RNA expression of the 90 mapped candidate genes in esophageal biopsies. Disease-dependent expression was observed for 22 of 59 candidates that met expression thresholds; 19 of the 22 genes were upregulated in the diseased state (Fig. 6). This reflected significant enrichment for differentially expressed genes among EoE risk candidates compared to genomic background (22/59 [37%] vs. 2,684/11,340 [24%], Fisher exact test p = 0.020), affirming that the mapping process produced disease-relevant genes.

Ingenuity Pathway Analysis of the 90 candidate risk genes revealed pathways enriched for inflammatory signaling pathways, including canonical Th1 and Th2 activation (*CD247*, *IL13*, *IL18R1*, *IL2*, *IL4*, *IL4R*, *IL5*, *IRF1*, *STAT6*, *TNFSF4*, *TSLP*) (Tables S6 and S11). Other pathways identified included IL-27 signaling, NOD1/2 signaling, and the regulators BCL6, TNFSF4, and EBI3 (IL-27 subunit beta). IL-27 has diverse roles in innate and adaptive immunity mediated in part through activation of JAK/STAT signaling in CD4 + T cells, whereby IL-27 can promote or suppress inflammation depending on context ^57^. Though NOD1/2 signaling is typically associated with pathogen recognition and innate immune responses, genetic variants in *NOD1* and *NOD2* have been associated with atopic dermatitis ^58–60^, suggesting a role in allergic inflammation. BCL6 is a transcriptional repressor that can compete for STAT DNA binding sites, suppressing STAT-dependent IL-4 responses, and is itself regulated by STATs ^61^. TNFSF4 is a TNF family cytokine that promotes T cell recruitment, proliferation, and cytokine production, with genetic polymorphisms that have been linked to allergic rhinitis ^62^. Further research is needed to determine whether these upstream regulators drive EoE pathology and whether they may be viable therapeutic targets.

### EoE genetic risk loci highlight disease-specific transcriptional mechanisms and cell types

To identify common transcriptional mechanisms that might be controlling multiple risk loci, enrichment for transcription factor genomic binding events (i.e., chromatin immunoprecipitation sequencing [ChIP-seq] peaks) at EoE risk loci was assessed using the Regulatory Element Locus Intersection (RELI) method ^63^. Transcription factors MYB (Th2 cells), STAT5A (CD4 + T cells), and STAT5B (CD8 + T cells) (Table S7) bound EoE genetic risk loci more than expected compared to a null model of accessible chromatin. C-MYB promotes the proliferation and differentiation of hematopoietic stem cells and may directly impact CD8 + T cell differentiation and survival ^64^, whereas STAT5 has well-known roles in inflammatory signaling, especially TSLP-induced Th2 signaling in EoE, mast cell activation in atopic dermatitis, IL-5 signal transduction in eosinophils, and activation of innate lymphoid cells (ILC2) ^42,65–67^. A previous study examining allelic transcriptional regulatory activity at variants across previously published EoE risk loci activity identified enrichment for GATA3 ^68^. When assessing all EoE risk loci identified in this study, we observed overlap with GATA3 ChIP-seq peaks at 16 risk variants spanning independent risk loci; however, enriched GATA3 ChIP-seq overlap was not robust to multiple testing correction (p-adjusted > 0.05).

**Figure 7:**
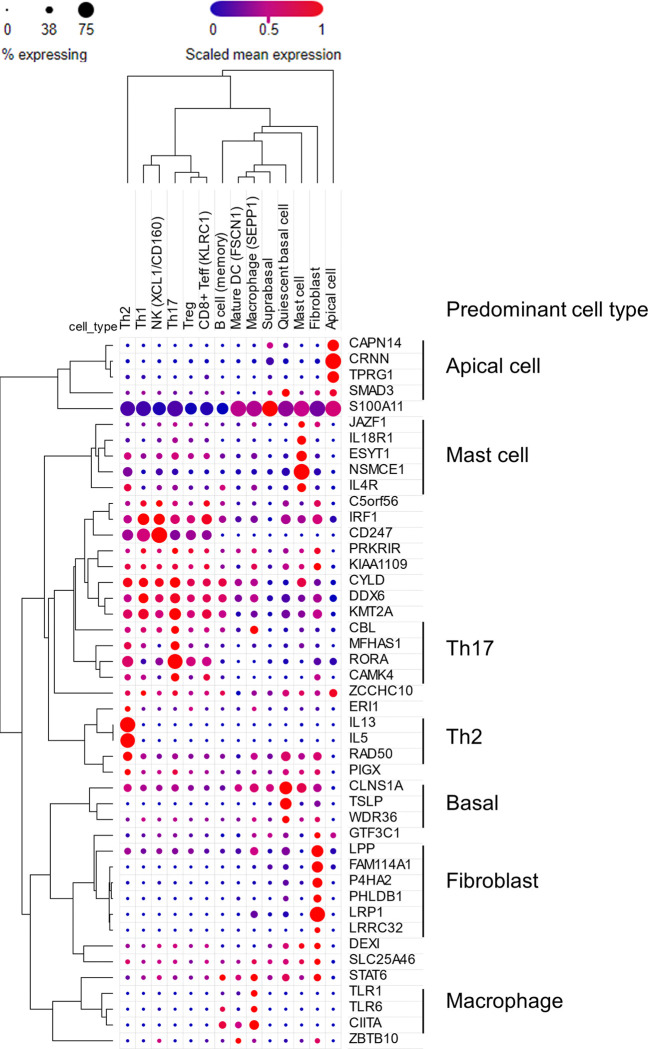
Cellular localization of EoE risk candidates. Dot plot showing cellular localization, as assessed by single-cell RNA sequencing, of 45/90 candidate genes expressed in esophageal biopsies (EoE n = 15, control n = 7; % expressing > 15% in at least one cell type). Dot size indicates the percent of cells in the cell type expressing each gene, and dot color is proportional to the mean of the per-cell expression (Broad Single-cell Portal, EoEBroad Single-cell Portal, EoE). DC, dendritic cell; NK, natural killer cell; Teff, effector T cell; Th, T helper cell; Treg, regulatory T cell.

To understand which cell types express the candidate risk genes in EoE, we interrogated a recently published single-cell RNA sequencing (scRNA-seq) dataset that identified 60 distinct cell states in the esophagus in the context of EoE ^69^. Forty-five of 90 candidates were expressed in biopsies in at least one cell type (Fig. 7 and Figure S7). Candidate genes were expressed across a diverse range of cell types, with 29 of 45 genes coalescing into distinct clusters of cellular localization. Seven genes were predominantly localized to the epithelium: apical (*CAPN14*, *CRNN*, *TPRG1*, *SMAD3*) and basal (*CLNS1A*, *TSLP*, *WDR36*), consistent with these genes’ roles in barrier function, external sensing, and initiation of inflammation ^70^. Six genes were predominantly expressed in fibroblasts (*LPP*, *FAM114A1*, *P4HA2*, *PHLDB1*, *LRP1*, *LRRC32*), suggesting a role for fibroblasts in sustaining inflammation and disrupting restoration of barrier integrity as seen in a model of atopic dermatitis ^71^ and recent studies implicating fibroblasts in EoE pathogenesis ^22,72^. Five genes predominantly localized to mast cells (*JAZF1*, *IL18R1*, *ESYT1*, *NSMCE1*, *IL4R*), consistent with increasing attention on mast cells as drivers of EoE pathology ^73^. Three genes were largely localized to macrophages (*TLR1*, *TLR6*, *CIITA*), suggesting a role for inappropriate or excessive activation of antigen-presenting cells in EoE pathogenesis ^74^. Numerous genes were broadly expressed in lymphocytes and group 2 ILCs (ILC2s), with some more specifically expressed in specific CD4 + T cell subsets. Three genes localized to ILC2s and Th2 cells (*IL13*, *IL5*, *RAD50*), consistent with the understanding of EoE as a Th2-driven atopic disease ^75^. Four genes localized to Th17 cells (*CBL*, *MFHAS1*, *RORA*, *CAMK4*), though many of these genes were also expressed to a lesser extent in Th2 cells. In atopic dermatitis, IL-17–producing Th17 cells have been postulated to be bystanders responding to general barrier dysfunction ^76^, though their association with EoE genetic susceptibility here suggests that they deserve further investigation.

## Discussion

Herein, we have advanced the understanding of the genetic architecture and underlying pathophysiology of EoE by expanding GWAS data and conducting the first multi-trait analysis of GWAS incorporating other atopic conditions, including asthma, allergic rhinitis, and atopic dermatitis. Through the collective examination of 1,757 individuals with EoE and 93,338 individuals with atopic disease, GWAS identified 11 variants across 8 risk loci; 3 of these loci were novel and included regions near classical immune regulators, such as *GATA3* and *IL4R*. The finding of a novel male-specific association at 12q24.31 (*SCARB2*) is consistent with the observed higher heritability of EoE in males that is established by familial EoE and twin studies ^77,78^ and a recent independent GWAS that identified 5 distinct sex-specific risk loci ^22^.

The multi-trait analysis of EoE and atopy GWAS (EoE-atopy-MTAG) demonstrates how the strong genetic correlations between EoE and other atopic diseases can be leveraged to identify new EoE susceptibility loci. Importantly, 33 independent loci were identified through MTAG analysis, 13 of which were novel in EoE. Beyond genetic risk loci, the MTAG approach implicated genes potentially important in EoE, including those mapped to the epidermal differentiation complex at 1q21, which contains the greatest density of genes dysregulated in the EoE transcriptome, such as *FLG*, *FLG2*, and *CRNN* ^79^. Despite the statistical testing bias limitations of the MTAG approach ^52^ and an imbalanced cohort size of EoE compared with the other atopic diseases, the MTAG approach employed in our study added valuable findings beyond the traditional GWAS approach. MTAG allowed us to leverage publicly available GWAS data (93,000 atopic individuals in the UKB) for the analysis of EoE susceptibility, illustrating MTAG’s utility for the study of less common diseases. MTAG-derived associations demonstrated high overlap with traditional EoE GWAS loci, verifying the approach. Moreover, the effect size (OR) for the MTAG-derived associations was substantially larger in EoE than other atopic diseases. The MTAG-derived PRS outperformed the conventional GWAS–derived PRS (EoE_1_ cohort), further validating the efficacy of the MTAG approach. Importantly, the MTAG approach did not replicate the 2p23 (*CAPN14*) EoE risk locus, consistent with the lack of association at this locus with other allergic diseases. MTAG complements but does not replace single-trait GWAS approaches as it will miss trait specific loci such as *CAPN14*. Collectively, our findings substantiate the hypothesis of a shared genetic basis for EoE and other atopic disease ^52^.

This study leveraged multiple mapping approaches together with single-trait and multi-trait GWAS to comprehensively map a total of 90 candidate EoE risk genes, greatly expanding the catalog of genes that may contribute to EoE disease susceptibility. Twenty-two of these candidate genes showed disease-dependent differential expression, orthogonally emphasizing potential contributions to EoE pathogenesis. Pathway enrichment analysis reinforced the central role of type 2 immunity in EoE pathogenesis. Genes in the NOD1/2 signaling pathway (e.g., *CYLD* and *NOD2*) and IL-27 regulatory pathways suggest broader immune regulation beyond type 2 responses, which may interact with microbial and environmental triggers. The EoE risk genes were expressed in a diverse set of cells, mainly coalescing into 7 distinct cellular clusters, including epithelial cells (basal and apical populations), fibroblasts, mast cells, myeloid cells, and polarized T helper subsets, especially Th2 and Th17 cells, the latter cells being previously underappreciated in EoE.

We observed enrichment of transcription factor genomic binding events for STAT5A/B and MYB, known regulators of Th2 cell activation and survival. STAT5 signaling, particularly in response to TSLP, has a crucial role in promoting Th2-mediated inflammation and eosinophilia, whereas MYB promotes T cell differentiation and hematopoietic cell proliferation. These findings highlight shared transcriptional circuits between genetic risk loci, providing opportunities for targeted interventions. Additional studies are warranted to comprehensively assess allelic transcriptional regulation at the expanded list of EoE risk loci.

A key outcome of this study is the development and validation of a PRS for EoE. Our previous study measured the increased effect size–weighted genetic burden of EoE risk variants for cases with EoE relative to controls but did not validate a PRS in an independent cohort ^24^. Herein, we validated the PRS findings identified in the EoE_1_ cohort with the EoE_2_ cohort, demonstrating robust performance: individuals in the top compared to bottom decile showed nearly 12-fold increased risk of developing EoE. For comparison, the top effect size EoE risk variant (rs77746069) showed only a 2-fold increased risk (Table 1A). Notably, the MTAG-derived PRS outperformed the EoE-specific GWAS-derived PRS model compared to PRS-EoE_GWAS_, substantiating the proposed value of MTAG for calculating ^52^ polygenic risk. Notably, the magnitude of the PRS-EoE_MTAG_ is robust compared with that of PRS of other immune-mediated, polygenic, allergic diseases (e.g., asthma), which have only one third to one half of the PRS effect size of PRS-EoE_MTAG_^80–82^). The relatively large PRS-EoE_MTAG_ is consistent with the larger sibling risk ratio seen in EoE compared with other atopic diseases ^77,78^. These findings facilitate future efforts to integrate PRS into risk stratification, early diagnosis, and precision medicine strategies for EoE.

In conclusion, this study expands the genetic landscape of EoE and highlights pathways central to its pathogenesis, including type 2 inflammation, epithelial barrier dysfunction, and innate immune activation. By integrating functional genomics and multi-trait analyses, we provide novel insights into the shared genetic architecture of EoE and other allergic diseases. The validated PRS underscores the potential for genetic risk assessment in clinical practice, while the identification of novel loci increases possibilities for future therapeutic targeting. Finally, an interactive tool (EGIDExpress) enables queries and visualize datasets, which provides a useful resource for the field of type 2 immunity. Together, these findings represent a significant step toward understanding, diagnosing, and treating EoE within the context of atopic disease.

## Methods

### GWAS participants

The study was approved by the Institutional Review Boards at Cincinnati Children’s Hospital Medical Center (CCHMC) and all participating sites that were part of the National Institutes of Health (NIH) Consortium of Food Allergy Research (CoFAR) ^50^ and the Consortium of Eosinophilic Gastrointestinal Disease Researchers (CEGIR) EoE Cohorts ^83,84^. Cases were confirmed by a physician as fulfilling the diagnostic criteria for EoE (peak eosinophil count ≥ 15 eosinophils / high-power field in esophageal biopsy sections) ^85^. A participant was considered to have atopy if they had a clinical history of atopic dermatitis, asthma, allergic rhinitis, and/or food allergy. Self-reported history of allergic rhinitis and atopic dermatitis was sufficient for diagnosis, when available.

Participants with EoE included 1,204 newly genotyped subjects who were included in EoE_1_ or EoE_2_ on the basis of genotype platform; EoE_1_ included those cases genotyped on the Illumina OMNI2.5 or OMNI5, whereas EoE_2_ included those cases genotyped on the Infinium Global Screening Array (GSA). Data from 553 subjects with EoE whose data were deposited in the database of Genotypes and Phenotypes (dbGaP) under accession phs000494.v1.p1 from a prior study ^19^ were included in EoE_1_ to further increase statistical power to identify new associations. For comparisons with EoE, atopic controls were randomly selected from the UKB dataset to equally represent atopic dermatitis, asthma, and allergic rhinitis (project 47377). External controls were from the University of Michigan Health and Retirement System (obtained from dbGAP accession phs000428.v1.p1), FECD-CIDR study (phs000421.v1.p1), KIDRISK study (phs001271.v1.p1), Framingham SNP Health Association Resource (phs000342.v21.p14), and Cincinnati Genomic Control Cohort ^86^. Participants in the UKB dataset were stratified by atopic status using the mapped international classification of diseases (ICD) diagnostic codes (Table S6) ^87^.

### Genome-wide genotyping

Genotyping was performed on both the Illumina OMNI-2.5 genotyping array (n = 480) as previously described ^19^ and the Infinium GSA (n = 724) according to the manufacturer’s protocol by the Regeneron Genetics Center ^88,89^.

### Genotyping quality control

Quality control for autosomal variants was performed in a stepwise process using a pipeline adapted from Marees *et. al* ^90^. Strand alignment was performed using the McCarthy Group Imputation Preparation and Checking Tool (https://www.well.ox.ac.uk/~wrayner/tools/). Genomic inflation and other relevant data for each dataset are summarized in Table S5.

### Relatedness and population stratification

Related individuals (2nd degree or closer) were excluded using GRAF-rel ^91^. This study was performed based on the availability of sufficient numbers of consented subjects of one ancestral group (European) to perform an informative analysis. Individuals with non-European ancestry were excluded using GRAF-pop ^92^.

### Genome-wide genotype imputation

Genotype imputation was performed using the TOPMed Imputation Server running Minimac4 1.5.7 with TOPMed r2 mixed ancestry reference panel ^93^. Imputed data were retained for variants with minor allele frequency exceeding 1% and variance ratio (r^2^) exceeding 0.8. Imputed data were mapped to SNPs using dbSNP b151.

### Genetic association analyses

Association analyses were performed in PLINKv2.0.

For all analyses, a lead genetic variant was defined as the top putatively independent association in an LD block (r^2^ < 0.1, based on the UK Biobank release 2b reference panel), whereas a risk locus was defined as the cluster of ≥ 1 lead variants within 250 kb. A novel risk locus was defined as containing ≥ 1 lead variant > 250 kb distant and linkage independent (r^2^ < 0.1) from previously published EoE risk variants in the GWAS catalog ^94^. For risk loci with multiple lead variants in the single-trait analysis of EoE, independent association was verified by conditional logistic regression analysis. Independent significant variants were defined by FUMA ^95^ and were used for LD expansion and candidate mapping.

### Cross-platform analyses

Cases and controls were genotyped on different platforms in the association studies EoE_2_, EoE_T_ (which contained EoE_2_), EoE vs. atopic controls, and EoE vs. nonatopic controls (Table S5). Potential batch effects were addressed as follows.

To identify and account for potential batch effects in EoE_2_ (EoE on the GSA, controls on Omni2.5/Omni5), variant calls were compared for 634 samples that were genotyped on both platforms. Two million nine-hundred thousand imputed variants (36%) that were not strictly concordant (p < 0.9) were excluded from most analyses, including tables and Manhattan plots, resulting in a genomic inflation (λ) factor of 1.06. The threshold was relaxed to p < 0.4 for Zoom plots to increase coverage in small windows where platform biases would not be expected to drive discovery of associated variants.

For EoE_T_, in the absence of variant data in EoE_2_, EoE_1_ variant data were used instead (reducing the sample size from 1,757 EoE to 1,033 EoE). For Manhattan plots, variants missing in EoE_2_ were excluded from analysis to avoid large inconsistencies in statistical power that would incorrectly imply differences in effect size. The final genomic inflation was 1.05 with discordant variants excluded.

For EoE vs. atopic and EoE vs. nonatopic controls (EoE on Omni2.5 and GSA, controls on UKB Axiom), 36,000 imputed variants were excluded that showed low concordance (p < 0.001) between control populations genotyped on Omni2.5 and UKB Axiom. Due to the high degree of population stratification observed between controls in the UKB study and controls in the other cohorts, the European ancestry cutoff in GRAF-pop was tightened to P_e_ > 0.99 from the standard threshold of P_e_ > 0.87, excluding ~ 60% of UKB participants that would typically be classified as being of European ancestry. Batch effects between GSA and Omni were handled as described with EoE_T_. The final genomic inflation was 1.13 for EoE vs. atopic and 1.11 for EoE vs. nonatopic controls. Associations within the UKB study, such as those that constituted the EoE-atopy-MTAG multi-trait analysis, did not compare cases and controls on different platforms and therefore did not require these adjustments.

### Multi-trait analysis of EoE and other atopic diseases

We performed multi-trait analysis of GWAS summary statistics using MTAG (v.1.0.8)^52^ to increase power for discovery of genetic loci associated with EoE. MTAG jointly analyzes multiple sets of GWAS summary statistics of genetically correlated traits to enhance statistical power ^52^. Regression coefficients (beta) and their standard errors were used as inputs for MTAG. The MTAG program aligned all alleles on the basis of different summary statistics and ensured that single-nucleotide polymorphisms (SNPs) were present in all datasets. SNPs that were not present in any dataset were removed. The final count of SNPs for MTAG analyses was 6,078,836.

### Candidate gene mapping

Independent significant variants from association analyses were mapped to genes using three methods: eQTL (esophageal mucosa in GTEx v8 ^96^, immune cells in the eQTL catalogue ^97^, and DICE ^98^), chromatin interaction (based on FANTOM5 project chromatin looping data in esophageal and immune cells ^99^), and position (within 10 kb of the transcribed region). Mapping was performed using the FUMA GWAS tool v1.5.0 with default parameters unless otherwise specified ^95^. The HLA region was excluded from mapping due to its complex LD structure.

### RNA sequencing

mRNA was isolated from the distal esophagus from patients with EoE with active disease and from non-EoE controls as previously described ^19,100,101^. EoE biopsies showed active disease pathology at the time when they were taken, and all patients had no glucocorticoid treatment at the time of biopsy. RNAseq acquiring 10 million mappable 75 base-pair reads from paired-end libraries was performed at the Genomics Sequencing Facility at CCHMC. Data were aligned using Ensembl ^102^ annotations as a guide for TopHat ^103^ using the default parameter settings. Expression analysis was performed by DESeq2 in BioWardrobe using the default parameter settings ^104,105^.

### Polygenic risk scores

PRS were calculated using PRS–continuous shrinkage (CS), a Bayesian polygenic prediction method that infers posterior effect sizes of genetic variants using GWAS summary statistics in the context of LD between variants as assessed on an external reference panel (i.e., the Phase 3 release of the 1,000 Genomes data) ^106^. The PRS training process used the Discovery GWAS summary statistics (MTAG) and the individual-level genotype data of the training GWAS data from a prior study ^19^ to tune the hyper-parameters of the prediction model using CS (auto mode) so that the pipeline automatically learned the sparseness of the genetic architecture from data and adjusted for the LD structure accordingly ^106^.

Confounding effects due to population stratification were adjusted using a linear regression model with the 10 principal components of ancestry in all participants^107^. After calculating a principal component–adjusted PRS, age and sex were used as covariates in a logistic regression fitting model implemented in R version 4.1.0.

PRS prediction accuracy and performance were assessed using OR per 1 standard deviation in logistic regression after accounting for covariates (10 principal components, age, and sex). To measure the clinical utility of EoE-PRS, we report the OR top decile of this distribution as a high polygenic score and report the increased odds of EoE by comparing the top decile to the bottom decile.

### Data availability in the interactive EGIDExpress

Data from this study can be accessed and filtered on the EGIDExpress GWAS app (https://egidexpress.research.cchmc.org/GWAS/). Variants that exhibit a putative association with EoE genetic susceptibility can be searched by SNP, band, or nearest gene to view their disease association (p-value and OR) in each cohort in the EoE susceptibility tab. MTAG analysis (p-value and beta) and the results of the component GWAS can be viewed in the EoE-atopy-MTAG tab. In the GWAS Viewer tab, results of GWAS for datasets EoE_1_ and EoE_2_ can be surveyed for any SNP manually entered. On each tab, LocusZoom, dbSNP, GTEx Portal, and GWAS Catalog external links are generated if they exist for the chosen variant. A tutorial for this tool is available: https://youtu.be/roPkMGzzLRM.

## Supplementary Material

This is a list of supplementary files associated with this preprint. Click to download.

240822Supplementarytablesv53.xlsx

Extendeddatafigures.docx

## Figures and Tables

**Table 1A.** Risk variants identified in the EoE-GWAS (Significant risk, p < 5×10^−8^)

								EoE_T_		EoE_1_		EoE_2_		EoE_1_ vs. non-atopic control		EoE_1_ vs. atopic control		EoE-atopy-MTAG	
Risk Locus	Category	Chr Band	Nearest Gene	ID	REF/ALT/A1	A1 CASE FREQ	A1 CTRL FREQ	OR	P	OR	P	OR	P	OR	P	OR	P	Beta	P
1	Known	2p23.1	*GALNT14*	rs77746069	G/**C/C**	0.04	0.02	2.14	1.24E-08	2.14	1.24E-08	-	-	2.16	2.84E-07	2.04	2.47E-06	-	-
1	Known	2p23.1	*CAPN14*	rs77569859	T/**C/C**	0.08	0.05	1.86	1.03E-19	2.04	3.02E-17	1.54	4.72E-04	1.78	1.52E-11	1.85	5.04E-13	0.051	4.39E-04
1	Known	2p23.1	*CAPN14*	rs11124250	**A**/G/**A**	0.54	0.47	1.35	1.36E-10	1.35	1.36E-10	-	-	1.38	4.29E-09	1.35	4.30E-08	0.015	1.28E-02
2	Known	5q22.1	*WDR36*	rs6594499	**C**/A/A	0.42	0.50	0.72	1.34E-19	0.67	9.54E-17	0.80	4.85E-04	0.77	1.37E-08	0.79	2.32E-07	−0.079	8.84E-38
3	Known	5q23.1	None	rs73782836	G/**A/A**	0.04	0.02	1.99	3.59E-08	1.99	3.59E-08	-	-	1.95	1.33E-05	2.01	5.24E-06	0.073	1.06E-03
10	Novel	8q21.13	*AC034114.2*	rs13274782	**A**/C**/A**	0.44	0.38	1.22	3.09E-08	1.27	2.59E-07	1.13	5.03E-02	1.27	1.57E-07	1.23	7.57E-06	0.042	4.07E-11
11	Novel	10p14	None	rs7898135	**C**/A/A	0.25	0.29	0.78	3.46E-09	0.76	4.75E-07	0.80	1.92E-03	0.73	1.56E-09	0.79	3.37E-06	−0.071	1.61E-25
13	Known	11q13.5	*EMSY*	rs61894547	C/**T/T**	0.07	0.05	1.55	1.40E-09	1.55	2.95E-06	1.50	1.16E-03	1.61	8.94E-08	1.34	6.81E-04	0.160	9.15E-29
13	Known	11q13.5	*EMSY*	rs7936323	G/**A/A**	0.54	0.48	1.26	2.04E-10	1.26	7.90E-07	1.31	1.70E-05	1.27	8.89E-08	0.87	1.80E-03	0.082	1.70E-40
20	Known	16p13.13	*CLEC16A*	rs12919828	**A**/G/G	0.20	0.25	0.73	3.15E-08	0.73	3.15E-08	-	-	0.76	3.55E-05	0.76	4.47E-05	−0.072	2.00E-24
21	Novel	16p12.1	*NSMCE1*	rs7195219	G/**A/A**	0.37	0.32	1.23	3.04E-08	1.16	1.96E-03	1.35	2.55E-06	1.22	2.08E-05	1.18	2.96E-04	0.032	1.40E-06

Eosinophilic esophagitis (EoE) risk alleles are **bolded**. Risk locus: Discrete 250-kb blocks ordered by chromosomal position; Category: Whether the variant overlap with previously published EoE risk loci; CHR: chromosome; REF: Reference allele; ALT: Alternate allele; A1: Effect allele; CTRL: control; FREQ: Allele frequency; OR: Odds ratio; MTAG, multi-trait analysis of genome-wide association studies; EoE_T_: The analysis of the merged EoE_1_ (EoE n = 1033) and EoE_2_ (EoE n = 724) cohorts.

**Table 1B.** EoE risk variants identified in the EoE-GWAS (Putative risk, 1×10^−6^ < p< 5×10^−8^)

							EoE_T_		EoE_1_		EoE_2_		EoE_1_ vs non-atopic control		EoE_1_ vs. atopic control		EoE-atopy-MTAG	
Risk Locus	Chr Band	Nearest Gene	ID	REF/ALT/A1	A1 CASE FREQ	A1 CTRL FREQ	OR	P	OR	P	OR	P	OR	P	OR	P	Beta	P
4	5q31.1	IL13	rs848	A/C/A	0.24	0.21	1.25	2.51E-07	1.26	2.30E-05	1.19	1.87E-02	1.28	6.37E-06	1.22	3.44E-04	0.059	1.18E-14
5	6q15	BACH2	rs5878119	G/GA/ GA	0.38	0.43	0.82	1.11E-07	0.77	1.06E-07	0.89	8.02E-02	0.83	8.72E-05	0.79	4.51E-07	-	-
6	7p14.1	INHBA	rs10225596	C/T/T	0.14	0.11	1.39	8.59E-07	1.39	8.59E-07	-	-	1.39	2.13E-05	1.30	7.22E-04	0.026	9.42E-03
7	7q35	EPHA1-AS1	rs117926980	G/C/C	0.05	0.03	1.55	3.91E-07	1.62	8.14E-06	1.47	1.48E-02	1.40	1.20E-03	1.33	5.58E-03	-	-
8	8p23.1	XKR6	rs10105377	A/G/G	0.42	0.47	0.82	7.30E-08	0.80	4.12E-06	0.84	4.45E-03	0.85	3.30E-04	0.84	1.36E-04	0.019	1.54E-03
9	8p11.21	None	rs186277585	T/G/G	0.03	0.01	1.88	1.80E-07	1.95	1.02E-05	1.75	9.54E-03	1.56	4.30E-03	1.48	9.39E-03	0.061	1.90E-02
12	10q23.33	MYOF	rs149220071	A/G/G	0.03	0.02	2.02	6.11E-07	2.02	6.11E-07	-	-	1.93	6.84E-05	2.09	5.23E-06	0.079	1.47E-03
14	11q23.3	None	rs11217045	C/A/A	0.16	0.21	0.73	5.29E-07	0.73	5.29E-07	-	-	0.74	3.76E-05	0.73	1.59E-05	0.046	1.37E-09
15	11q25	OPCML	rs61910353	C/T/T	0.15	0.12	1.29	6.42E-07	1.21	3.87E-03	1.45	2.07E-05	1.23	1.74E-03	1.28	9.92E-05	0.025	8.53E-03
16	12q24.31	SCARB1	rs34399476	CT/C/CT	0.19	0.15	1.26	8.04E-07	1.18	6.62E-03	1.42	1.20E-05	1.22	9.74E-04	1.18	4.41E-03	-	-
17	13q14.3	DLEU1	rs806312	A/G/G	0.41	0.36	1.20	5.41E-07	1.18	4.18E-04	1.26	2.40E-04	1.27	1.42E-07	1.13	5.46E-03	0.023	3.58E-04
18	14q31.3	FLRT2	rs146927493	A/C/C	0.02	0.01	1.88	6.38E-07	1.68	1.85E-03	2.20	4.60E-04	1.61	1.84E-03	1.51	6.21E-03	0.052	5.29E-02
19	15q22.2	RORA	rs1817479	G/C/C	0.11	0.14	0.75	4.58E-07	0.73	2.62E-05	0.78	7.71E-03	0.74	2.02E-05	0.75	6.91E-05	-	-
22	18q11.2	AQP4-AS1	rs2082098	A/C/A	0.41	0.36	1.26	9.94E-07	1.26	9.94E-07	-	-	1.26	3.49E-05	1.22	3.24E-04	0.007	2.71E-01

Eosinophilic esophagitis (EoE) risk alleles are bolded. Risk locus: Discrete 250-kb blocks ordered by chromosomal position; Chr: chromosome; REF: Reference allele; ALT: Alternate allele; A1: Effect allele; CTRL: control; FREQ: Allele frequency; OR: Odds ratio; MTAG, multi-trait analysis of genome-wide association studies; EoE_T_: The analysis of the merged EoE_1_ (EoE n = 1033) and EoE_2_ (EoE n = 724) cohorts.

**Table 2A.** Novel EoE risk variants identified in the multi-trait analysis of EoE_1_ and other atopic diseases (Significant risk, MTAG p < 5×10^−8^ and EoE_1_ p < 0.05)

							EoE-atopy-MTAG		EoE_1_		UKB Asthma		UKB Rhinitis		UKB Atopic dermatitis	
Risk Locus	Chr Band	Nearest Gene	ID	REF/ALT/A1	A1 CASE FREQ	A1 CTRL FREQ	Beta	P	OR	P	OR	P	OR	P	OR	P
1	1q21.3	*AL589986.2*	rs12123821	C**/T/T**	0.06	0.05	0.092	1.71E-10	1.29	1.27E-02	1.16	1.24E-17	1.10	2.66E-06	1.47	2.92E-53
3	1q24.2	*CD247*	rs2995089	**G**/A/A	0.36	0.38	−0.051	6.80E-16	0.85	5.66E-04	0.94	1.24E-13	0.94	4.76E-13	0.96	3.21E-03
4	1q25.1	*TNFSF4*	rs7518129	A/**G/G**	0.37	0.33	0.042	1.89E-10	1.14	8.25E-03	1.04	1.41E-05	1.05	1.09E-08	1.06	3.20E-06
8	3q28	*LPP*	rs12639588	A/**G/G**	0.50	0.45	0.039	4.30E-10	1.22	2.52E-05	1.04	2.26E-08	1.04	5.09E-06	1.03	2.83E-02
9	3q29	*FBXO45*	rs112336433	**C**/T/T	0.05	0.06	−0.077	1.41E-09	0.79	2.60E-02	0.91	5.91E-09	0.91	1.03E-07	0.92	1.70E-03
11	4q27	*KIAA1109*	rs6534338	**T**/C/**T**	0.32	0.30	0.039	8.19E-09	1.13	1.06E-02	1.05	1.25E-07	1.06	2.27E-09	1.04	7.36E-03
11	4q27	*KIAA1109*	rs7688384	**C**/T/T	0.31	0.32	−0.060	1.52E-19	0.86	2.43E-03	0.94	2.75E-12	0.91	4.10E-23	0.95	4.62E-04
17	7p15.1	*JAZF1*	rs10245867	G/**T/T**	0.36	0.34	0.036	1.91E-08	1.10	4.26E-02	1.05	1.90E-09	1.04	1.66E-06	1.02	1.68E-01
18	8p23.1	*PRAG1*	rs793728	**A**/G/G	0.36	0.40	−0.036	9.70E-09	0.87	5.84E-03	0.96	1.97E-08	0.98	2.57E-02	0.97	4.44E-02
20	9p24.1	*RANBP6*	rs340934	**G**/T/**G**	0.21	0.20	−0.048	6.39E-10	1.13	3.71E-02	0.90	1.62E-26	0.94	2.40E-07	0.98	2.93E-01
24	11q23.3	*CXCR5*	rs11217074	**T**/C/C	0.17	0.20	−0.051	3.34E-11	0.76	1.84E-05	0.95	7.42E-08	0.94	1.74E-07	0.95	2.63E-03
28	14q24.1	*RAD51B*	rs1950897	**C**/T/**C**	0.34	0.30	0.046	1.35E-11	1.18	9.54E-04	1.06	1.25E-11	1.05	1.22E-06	1.05	3.46E-04
33	16q12.1	*CYLD*	rs12324931	**A**/C/C	0.06	0.05	0.083	1.03E-08	1.35	2.06E-03	1.08	1.20E-05	1.10	1.36E-06	1.10	1.74E-03
35	19q13.11	*SLC7A10*	rs117710327	**C**/A/A	0.04	0.07	−0.106	1.63E-17	0.61	1.94E-05	0.89	1.36E-12	0.89	3.29E-10	0.92	2.20E-03

Eosinophilic esophagitis (EoE) risk alleles are **bolded**. Risk locus: Discrete 250-kb blocks ordered by chromosomal position; Chr: chromosome; REF: Reference allele; ALT: Alternate allele; A1: Effect allele; CTRL: control; FREQ: Allele frequency; OR: Odds ratio; MTAG, multi-trait analysis of genome-wide association studies; UKB: United Kingdom Biobank; EoE_T_: The analysis of the merged EoE_1_ (EoE n = 1033) and EoE_2_ (EoE n = 724) cohorts.

**Table 2B.** Other EoE risk variants identified in the multi-trait analysis of EoE_1_ and other atopic diseases (Significant risk, MTAG p < 5×10^−8^ and EoE_1_ p < 0.05)

								EoE-atopy-MTAG		EoE_1_		UKB Asthma		UKB Rhinitis		UKB Atopic dermatitis	
Risk Locus	Category	Chr Band	Nearest Gene	ID	REF/ALT/A1	A1 CASE FREQ	A1 CTRL FREQ	Beta	P	OR	P	OR	P	OR	P	OR	P
13	Known	5q22.1	WDR36	rs6594499	C/A/A	0.42	0.50	−0.079	8.84E-38	0.67	9.54E-17	0.91	6.29E-34	0.91	2.70E-28	0.96	4.79E-03
13	Proximal	5q22.1	CAMK4	rs62376842	A/C/C	0.16	0.21	−0.043	1.90E-08	0.73	7.03E-07	0.95	8.78E-07	0.95	1.15E-06	0.98	3.32E-01
13	Proximal	5q22.1	CAMK4	rs10478040	C/T/T	0.04	0.05	−0.083	3.74E-09	0.75	1.78E-02	0.91	1.35E-07	0.89	3.32E-08	0.95	8.24E-02
14	Proximal	5q31.1	IL13	rs848	A/C/A	0.24	0.21	0.059	1.18E-14	1.26	2.30E-05	1.10	1.45E-22	1.04	8.58E-04	1.14	1.32E-16
15	Known	6q15	BACH2	rs905670	G/A/A	0.30	0.33	−0.056	3.36E-18	0.79	3.88E-06	0.92	5.22E-27	0.95	1.49E-07	0.94	8.70E-07
19	EoE_T_	8q21.13	AC034114.2	rs13272328	T/C/T	0.41	0.36	0.046	7.42E-13	1.27	3.39E-07	1.06	3.86E-14	1.04	2.96E-06	1.04	2.82E-03
22	Proximal	10p14	AC044784.2	rs12253380	A/G/G	0.05	0.06	−0.077	5.68E-09	0.76	1.31E-02	0.90	5.46E-09	0.91	1.71E-06	0.95	7.52E-02
22	Proximal	10p14	None	rs4582874	T/C/C	0.15	0.14	0.055	1.22E-09	1.16	2.42E-02	1.08	6.66E-14	1.06	6.60E-06	1.03	1.11E-01
22	EoE_T_	10p14	None	rs7898135	C/A/A	0.25	0.29	−0.071	1.61E-25	0.76	4.75E-07	0.90	2.00E-39	0.93	1.75E-13	0.96	1.76E-03
22	Proximal	10p14	None	rs1444789	T/C/C	0.21	0.19	0.076	1.47E-21	1.21	6.58E-04	1.13	1.99E-35	1.08	3.39E-12	1.06	2.76E-04
22	Proximal	10p14	None	rs72784452	C/T/T	0.07	0.08	−0.072	8.81E-10	0.75	2.44E-03	0.87	1.77E-19	0.95	1.10E-03	0.94	1.40E-02
23	Known	11q13.5	EMSY	rs7936312	G/T/T	0.54	0.48	0.082	1.37E-40	1.26	8.71E-07	1.10	3.17E-36	1.11	4.21E-33	1.13	4.33E-22
23	Known	11q13.5	EMSY	rs55646091	G/A/A	0.07	0.05	0.161	4.92E-29	1.51	1.46E-05	1.21	2.40E-29	1.21	4.15E-23	1.25	3.14E-16
27	Proximal	12q13.3	STAT6	rs3024971	T/G/G	0.09	0.11	−0.086	3.46E-18	0.67	2.70E-06	0.90	5.23E-17	0.91	1.64E-11	0.93	5.82E-04
27	Known	12q13.3	STAT6	rs167769	C/T/T	0.40	0.38	0.044	1.94E-12	1.10	4.80E-02	1.07	3.59E-18	1.05	3.96E-09	1.02	6.41E-02
29	Known	15q22.2	RORA	rs10519067	G/A/A	0.10	0.13	−0.069	3.41E-14	0.74	6.54E-05	0.94	2.62E-08	0.92	1.38E-11	0.98	3.33E-01
30	Known	15q22.33	SMAD3	rs56375023	G/A/A	0.26	0.24	0.071	5.45E-23	1.16	6.46E-03	1.12	1.37E-37	1.10	1.93E-22	1.06	2.57E-04
31	Known	16p13.13	CLEC16A	rs36045143	A/G/G	0.20	0.25	−0.072	1.53E-24	0.73	4.17E-08	0.91	2.09E-24	0.92	4.20E-15	0.90	5.14E-12
32	Proximal	16p12.1	IL4R	rs3785356	C/T/T	0.33	0.29	0.045	4.22E-11	1.16	2.80E-03	1.07	1.06E-14	1.04	3.48E-06	1.02	1.16E-01

Eosinophilic esophagitis (EoE) risk alleles are bolded. Category: Whether the variant overlaps previously published EoE risk loci (Known), is adjacent to or in weak linkage disequilibrium (LD) with known loci (Proximal), or was first identified in EoE_T_ (combined EoE_1_ and EoE_2_); Risk locus: Discrete 250-kb blocks ordered by chromosomal position; Chr: Chromosome; REF: Reference allele; ALT: Alternate allele; A1: Effect allele; CTRL: Control; FREQ: Allele frequency; OR: Odds ratio; MTAG, multi-trait analysis of genome-wide association studies; UKB: United Kingdom Biobank.

**Table 3:** EoE candidate genes mapped by source and method

Association study / mapping method	Total number of genes	eQTL	Chromatin interaction	Position (+/− 10 kb)
EoE vs. population control (EoE_T_)	26	10	8	18
EoE vs. atopic control	10	7	2	8
EoE vs. non-atopic control	18	10	2	13
EoE-atopy-MTAG	86	40	17	63
**Total number of genes**	**90** *	**41** *	**17** *	**67** *

Ninety eosinophilic esophagitis (EoE) candidate genes were mapped to risk loci identified from EoE_T_, EoE vs. atopic controls, EoE vs. nonatopic controls, and EoE-atopy-MTAG analyses by expression quantitative trait locus (eQTL), chromatin interaction, or position (see Table S2). MTAG, multi-trait analysis of genome-wide association studies.

* The total number of genes is a summation of non-redundant genes from each analysis.

## References

[1] Furuta GT, Katzka DA (2015) Eosinophilic Esophagitis. N Engl J Med 373:1640–1648 26488694 10.1056/NEJMra1502863 PMC4905697

[2] Abonia JP, Rothenberg ME (2012) Eosinophilic esophagitis: rapidly advancing insights. Annu Rev Med 63, 421 – 34 22034864 10.1146/annurev-med-041610-134138

[3] Davis BP, Rothenberg ME (2016) Mechanisms of Disease of Eosinophilic Esophagitis. Annu Rev Pathol 11:365–393 26925500 10.1146/annurev-pathol-012615-044241 PMC4918086

[4] Simon D (2016) Eosinophilic esophagitis is characterized by a non-IgE-mediated food hypersensitivity. Allergy 71, 611 – 20 26799684 10.1111/all.12846

[5] Warners MJ, Vlieg-Boerstra BJ, Bredenoord AJ (2015) Elimination and elemental diet therapy in eosinophilic oesophagitis. Best Pract Res Clin Gastroenterol 29:793–803 26552778 10.1016/j.bpg.2015.06.013

[6] Thel HL, Anderson C, Xue AZ, Jensen ET, Dellon ES (2024) Prevalence and costs of eosinophilic esophagitis in the United States. Clin Gastroenterol Hepatol10.1016/j.cgh.2024.09.031 PMC11761390 39486752

[7] Hirano I (2020) Efficacy of Dupilumab in a Phase 2 Randomized Trial of Adults With Active Eosinophilic Esophagitis. Gastroenterology 158:111–122e10 31593702 10.1053/j.gastro.2019.09.042

[8] Zuo L (2010) IL-13 induces esophageal remodeling and gene expression by an eosinophil-independent, IL-13R alpha 2-inhibited pathway. J Immunol 185:660–669 20543112 10.4049/jimmunol.1000471 PMC3746758

[9] Muir AB (2022) Esophageal remodeling in eosinophilic esophagitis: Relationships to luminal captured biomarkers of inflammation and periostin. J Allergy Clin Immunol 150:649–656e5 35405206 10.1016/j.jaci.2022.03.022 PMC10367933

[10] Nhu QM, Aceves SS (2023) Current state of biologics in treating eosinophilic esophagitis. Ann Allergy Asthma Immunol 130:15–20 36243282 10.1016/j.anai.2022.10.004

[11] Ptaschinski C, Zhu D, Fonseca W, Lukacs NW (2023) Stem cell factor inhibition reduces Th2 inflammation and cellular infiltration in a mouse model of eosinophilic esophagitis. Mucosal Immunol 16:727–739 37557983 10.1016/j.mucimm.2023.07.006 PMC10680063

[12] Racca F (2021) Type 2 Inflammation in Eosinophilic Esophagitis: From Pathophysiology to Therapeutic Targets. Front Physiol 12:815842 35095572 10.3389/fphys.2021.815842 PMC8790151

[13] Rochman M, Azouz NP, Rothenberg ME (2018) Epithelial origin of eosinophilic esophagitis. J Allergy Clin Immunol 142:10–23 29980278 10.1016/j.jaci.2018.05.008 PMC8034427

[14] Muir AB, Wang JX, Nakagawa H (2019) Epithelial-stromal crosstalk and fibrosis in eosinophilic esophagitis. J Gastroenterol 54:10–18 30101408 10.1007/s00535-018-1498-3 PMC6314980

[15] Chang JW, Jensen ET, Dellon ES (2022) Nature with Nurture: the Role of Intrinsic Genetic and Extrinsic Environmental Factors on Eosinophilic Esophagitis. Curr Allergy Asthma Rep 22:163–170 36190688 10.1007/s11882-022-01042-1 PMC10838151

[16] Dowling PJ, Neuhaus H, Polk BI (2019) The Role of the Environment in Eosinophilic Esophagitis. Clin Rev Allergy Immunol 57:330–339 30032346 10.1007/s12016-018-8697-9

[17] Rochman M, Klinger AM, Caldwell JM, Sadovsky Y, Rothenberg ME (2024) Amniotic fluid modifies esophageal epithelium differentiation and inflammatory responses. Am J Physiol Gastrointest Liver Physiol 327:G629–G639 39189791 10.1152/ajpgi.00197.2024 PMC11559652

[18] Rothenberg ME (2010) Common variants at 5q22 associate with pediatric eosinophilic esophagitis. Nat Genet 42:289–291 20208534 10.1038/ng.547 PMC3740732

[19] Kottyan LC (2014) Genome-wide association analysis of eosinophilic esophagitis provides insight into the tissue specificity of this allergic disease. Nat Genet 46:895–900 25017104 10.1038/ng.3033 PMC4121957

[20] Sleiman PM (2014) GWAS identifies four novel eosinophilic esophagitis loci. Nat Commun 5:5593 25407941 10.1038/ncomms6593 PMC4238044

[21] Kottyan LC (2019) Genetic variants at the 16p13 locus confer risk for eosinophilic esophagitis. Genes Immun 20:281–292 29904099 10.1038/s41435-018-0034-z PMC6286696

[22] Chang X (2022) A genome-wide association meta-analysis identifies new eosinophilic esophagitis loci. J Allergy Clin Immunol 149:988–998 34506852 10.1016/j.jaci.2021.08.018 PMC9579995

[23] Gautam Y (2023) Genome-wide admixture and association analysis identifies African ancestry-specific risk loci of eosinophilic esophagitis in African Americans. J Allergy Clin Immunol 151:1337–1350 36400179 10.1016/j.jaci.2022.09.040 PMC10164699

[24] Kottyan LC (2021) Replication and meta-analyses nominate numerous eosinophilic esophagitis risk genes. J Allergy Clin Immunol 147:255–266 33446330 10.1016/j.jaci.2020.10.018 PMC8082436

[25] Sleiman PM, March M, Hakonarson H (2015) The genetic basis of eosinophilic esophagitis. Best Pract Res Clin Gastroenterol 29:701–707 26552769 10.1016/j.bpg.2015.09.003

[26] Martin LJ (2018) Eosinophilic esophagitis (EoE) genetic susceptibility is mediated by synergistic interactions between EoE-specific and general atopic disease loci. J Allergy Clin Immunol 141:1690–1698 29129581 10.1016/j.jaci.2017.09.046 PMC6016040

[27] Waage J (2018) Genome-wide association and HLA fine-mapping studies identify risk loci and genetic pathways underlying allergic rhinitis. Nat Genet 50:1072–1080 30013184 10.1038/s41588-018-0157-1 PMC7068780

[28] Astle WJ (2016) The Allelic Landscape of Human Blood Cell Trait Variation and Links to Common Complex Disease. Cell 167, 1415–1429 e19 27863252 10.1016/j.cell.2016.10.042 PMC5300907

[29] Kim SY (2021) Genome-wide association study identifies BTNL2 associated with atopic asthma in children. Med (Baltim) 100:e27626 10.1097/MD.0000000000027626 PMC8568460 34871226

[30] Lee SU, Maeda T (2012) POK/ZBTB proteins: an emerging family of proteins that regulate lymphoid development and function. Immunol Rev 247:107–119 22500835 10.1111/j.1600-065X.2012.01116.x PMC3334328

[31] Zheng X (2024) Unveiling genetic links between gut microbiota and asthma: a Mendelian randomization. Front Microbiol 15:1448629 39372270 10.3389/fmicb.2024.1448629 PMC11449699

[32] Sottile G (2019) An association analysis to identify genetic variants linked to asthma and rhino-conjunctivitis in a cohort of Sicilian children. Ital J Pediatr 45:16 30646946 10.1186/s13052-019-0603-4 PMC6334451

[33] Chen X (2022) Association of the IL-4R Q576R Polymorphism with Pediatric Asthma: a meta-analysis. Afr Health Sci 22:307–316 36910341 10.4314/ahs.v22i3.32 PMC9993270

[34] Yang B (2023) The IL-4Ralpha Q576R polymorphism is associated with increased severity of atopic dermatitis and exaggerates allergic skin inflammation in mice. J Allergy Clin Immunol 151, 1296–1306 e7 36690254 10.1016/j.jaci.2023.01.011 PMC10164706

[35] Davis BP (2016) Eosinophilic esophagitis-linked calpain 14 is an IL-13-induced protease that mediates esophageal epithelial barrier impairment. JCI Insight 1:e86355 27158675 10.1172/jci.insight.86355 PMC4855700

[36] Litosh VA (2017) Calpain-14 and its association with eosinophilic esophagitis. J Allergy Clin Immunol 139:1762–1771e7 28131390 10.1016/j.jaci.2016.09.027 PMC5461191

[37] Miller DE (2019) Genetic, Inflammatory, and Epithelial Cell Differentiation Factors Control Expression of Human Calpain-14. G3 (Bethesda) 9:729–736 30626591 10.1534/g3.118.200901 PMC6404614

[38] Ferreira MAR (2020) Age-of-onset information helps identify 76 genetic variants associated with allergic disease. PLoS Genet 16:e1008725 32603359 10.1371/journal.pgen.1008725 PMC7367489

[39] Sakaue S (2021) A cross-population atlas of genetic associations for 220 human phenotypes. Nat Genet 53:1415–1424 34594039 10.1038/s41588-021-00931-x PMC12208603

[40] Corren J, Ziegler SF (2019) TSLP: from allergy to cancer. Nat Immunol 20:1603–1609 31745338 10.1038/s41590-019-0524-9

[41] Cianferoni A, Spergel J (2014) The importance of TSLP in allergic disease and its role as a potential therapeutic target. Expert Rev Clin Immunol 10:1463–1474 25340427 10.1586/1744666X.2014.967684 PMC4332833

[42] Rochman Y (2023) TSLP shapes the pathogenic responses of memory CD4(+) T cells in eosinophilic esophagitis. Sci Signal 16:eadg6360 37699081 10.1126/scisignal.adg6360 PMC10602003

[43] Fahey LM (2018) EMSY is increased and activates TSLP & CCL5 expression in eosinophilic esophagitis. Pediatr Allergy Immunol 29:565–568 29663593 10.1111/pai.12907

[44] Vuckovic D (2020) The Polygenic and Monogenic Basis of Blood Traits and Diseases. Cell 182:1214–1231e11 32888494 10.1016/j.cell.2020.08.008 PMC7482360

[45] Olafsdottir TA (2020) Eighty-eight variants highlight the role of T cell regulation and airway remodeling in asthma pathogenesis. Nat Commun 11:393 31959851 10.1038/s41467-019-14144-8 PMC6971247

[46] Johansson A, Rask-Andersen M, Karlsson T, Ek WE (2019) Genome-wide association analysis of 350 000 Caucasians from the UK Biobank identifies novel loci for asthma, hay fever and eczema. Hum Mol Genet 28:4022–4041 31361310 10.1093/hmg/ddz175 PMC6969355

[47] Daya M (2021) Multiethnic genome-wide and HLA association study of total serum IgE level. J Allergy Clin Immunol 148:1589–1595 34536413 10.1016/j.jaci.2021.09.011 PMC8665111

[48] Franke A (2010) Genome-wide meta-analysis increases to 71 the number of confirmed Crohn’s disease susceptibility loci. Nat Genet 42:1118–1125 21102463 10.1038/ng.717 PMC3299551

[49] Kottyan LC, Parameswaran S, Weirauch MT, Rothenberg ME, Martin LJ (2020) The genetic etiology of eosinophilic esophagitis. J Allergy Clin Immunol 145:9–15 31910986 10.1016/j.jaci.2019.11.013 PMC6984394

[50] Chehade M (2018) Phenotypic Characterization of Eosinophilic Esophagitis in a Large Multicenter Patient Population from the Consortium for Food Allergy Research. J Allergy Clin Immunol Pract 6:1534–1544e5 30075341 10.1016/j.jaip.2018.05.038 PMC6132253

[51] Mohammad AA (2017) Prevalence of atopic comorbidities in eosinophilic esophagitis: A case-control study of 449 patients. J Am Acad Dermatol 76:559–560 28212761 10.1016/j.jaad.2016.08.068

[52] Turley P (2018) Multi-trait analysis of genome-wide association summary statistics using MTAG. Nat Genet 50:229–237 29292387 10.1038/s41588-017-0009-4 PMC5805593

[53] Abhishek S, Palamadai Krishnan S (2016) Epidermal Differentiation Complex: A Review on Its Epigenetic Regulation and Potential Drug Targets. Cell J 18:1–6 27054112 10.22074/cellj.2016.3980 PMC4819378

[54] Politi E (2017) Filaggrin and Periostin Expression Is Altered in Eosinophilic Esophagitis and Normalized With Treatment. J Pediatr Gastroenterol Nutr 65:47–52 28644349 10.1097/MPG.0000000000001419

[55] Wu L (2018) Filaggrin and tight junction proteins are crucial for IL-13-mediated esophageal barrier dysfunction. Am J Physiol Gastrointest Liver Physiol 315:G341–G350 29746170 10.1152/ajpgi.00404.2017

[56] Yu H (2022) HDL and Scavenger Receptor Class B Type I (SRBI). Adv Exp Med Biol 1377:79–93 35575922 10.1007/978-981-19-1592-5_6

[57] Aparicio-Siegmund S, Garbers C (2015) The biology of interleukin-27 reveals unique pro- and anti-inflammatory functions in immunity. Cytokine Growth Factor Rev 26:579–586 26195434 10.1016/j.cytogfr.2015.07.008

[58] Caruso R, Warner N, Inohara N, Nunez G (2014) NOD1 and NOD2: signaling, host defense, and inflammatory disease. Immunity 41:898–908 25526305 10.1016/j.immuni.2014.12.010 PMC4272446

[59] Kabesch M (2003) Association between polymorphisms in caspase recruitment domain containing protein 15 and allergy in two German populations. J Allergy Clin Immunol 111:813–817 12704363 10.1067/mai.2003.1336

[60] Weidinger S (2005) Association of NOD1 polymorphisms with atopic eczema and related phenotypes. J Allergy Clin Immunol 116:177–184 15990792 10.1016/j.jaci.2005.02.034

[61] Arima M, Fukuda T, Tokuhisa T (2008) Role of the Transcriptional Repressor BCL6 in Allergic Response and Inflammation. World Allergy Organ J 1:115–122 23282478 10.1097/WOX.0b013e31817dc522 PMC3651038

[62] Shen Y (2017) Association between TNFSF4 and BLK gene polymorphisms and susceptibility to allergic rhinitis. Mol Med Rep 16:3224–3232 28713926 10.3892/mmr.2017.6954 PMC5547929

[63] Harley JB (2018) Transcription factors operate across disease loci, with EBNA2 implicated in autoimmunity. Nat Genet 50:699–707 29662164 10.1038/s41588-018-0102-3 PMC6022759

[64] Gautam S (2019) The transcription factor c-Myb regulates CD8(+) T cell stemness and antitumor immunity. Nat Immunol 20:337–349 30778251 10.1038/s41590-018-0311-z PMC6489499

[65] Ando T (2014) Critical role for mast cell Stat5 activity in skin inflammation. Cell Rep 6:366–376 24412367 10.1016/j.celrep.2013.12.029 PMC4329986

[66] Morot J (2023) Hyperactivation of the JAK2/STAT5 Signaling Pathway and Evaluation of Baricitinib Treatment Among Patients With Eosinophilic Cellulitis. JAMA Dermatol 159:820–829 37342057 10.1001/jamadermatol.2023.1651 PMC10285679

[67] Kato A (2019) Group 2 Innate Lymphoid Cells in Airway Diseases. Chest 156:141–149 31082387 10.1016/j.chest.2019.04.101 PMC7118243

[68] Shook MS (2024) Systematic identification of genotype-dependent enhancer variants in eosinophilic esophagitis. Am J Hum Genet 111:280–294 38183988 10.1016/j.ajhg.2023.12.008 PMC10870143

[69] Ding J (2024) An esophagus cell atlas reveals dynamic rewiring during active eosinophilic esophagitis and remission. Nat Commun 15:3344 38637492 10.1038/s41467-024-47647-0 PMC11026436

[70] Rodriguez-Lopez EM, Hill DA (2024) Eosinophilic gastrointestinal disorders and the role for the epithelium in pathogenesis and treatment. Curr Opin Pediatr 36:668–673 39319691 10.1097/MOP.0000000000001406

[71] Berroth A (2013) Role of fibroblasts in the pathogenesis of atopic dermatitis. J Allergy Clin Immunol 131:1547–1554 23582515 10.1016/j.jaci.2013.02.029

[72] Jumabay M (2024) Eosinophilic esophagitis drives tissue fibroblast regenerative programs toward pathologic dysfunction. J Allergy Clin Immunol S0091–6749 10.1016/j.jaci.2024.11.028 PMC11980045 39617290

[73] Bolton SM (2020) Mast Cell Infiltration Is Associated With Persistent Symptoms and Endoscopic Abnormalities Despite Resolution of Eosinophilia in Pediatric Eosinophilic Esophagitis. Am J Gastroenterol 115:224–233 31913192 10.14309/ajg.0000000000000474 PMC7491279

[74] Barchi A (2024) From Pathogenesis to Treatment: Targeting Type-2 Inflammation in Eosinophilic Esophagitis. Biomolecules 14 10.3390/biom14091080 PMC11429508 39334846

[75] Oliva S, Azouz NP, Stronati L, Rothenberg ME (2020) Recent advances in potential targets for eosinophilic esophagitis treatments. Expert Rev Clin Immunol 16:421–428 32163308 10.1080/1744666X.2020.1742110

[76] Sugaya M (2020) The Role of Th17-Related Cytokines in Atopic Dermatitis. Int J Mol Sci 21 10.3390/ijms21041314 PMC7072946 32075269

[77] Collins MH (2008) Clinical, pathologic, and molecular characterization of familial eosinophilic esophagitis compared with sporadic cases. Clin Gastroenterol Hepatol 6:621–629 18434257 10.1016/j.cgh.2008.01.004 PMC2701188

[78] Alexander ES (2014) Twin and family studies reveal strong environmental and weaker genetic cues explaining heritability of eosinophilic esophagitis. J Allergy Clin Immunol 134:1084–1092e1 25258143 10.1016/j.jaci.2014.07.021 PMC4253562

[79] Blanchard C (2010) Coordinate interaction between IL-13 and epithelial differentiation cluster genes in eosinophilic esophagitis. J Immunol 184:4033–4041 20208004 10.4049/jimmunol.0903069 PMC3807813

[80] Dapas M (2023) Revealing polygenic pleiotropy using genetic risk scores for asthma. HGG Adv 4:100233 37663543 10.1016/j.xhgg.2023.100233 PMC10474095

[81] Namjou B (2022) Multiancestral polygenic risk score for pediatric asthma. J Allergy Clin Immunol 150:1086–1096 35595084 10.1016/j.jaci.2022.03.035 PMC9643615

[82] Stikker B (2024) Epigenomic partitioning of a polygenic risk score for asthma reveals distinct genetically driven disease pathways. Eur Respir J 64 10.1183/13993003.02059-2023 PMC11358516 38901884

[83] Furuta GT (2024) Building and implementing a research infrastructure for eosinophilic gastrointestinal diseases. J Allergy Clin Immunol 153:1536–1539 38849187 10.1016/j.jaci.2024.04.014 PMC11550568

[84] Aceves S (2020) Advancing patient care through the Consortium of Eosinophilic Gastrointestinal Disease Researchers (CEGIR). J Allergy Clin Immunol 145:28–37 31758958 10.1016/j.jaci.2019.11.012 PMC6981250

[85] Dellon ES (2018) Updated International Consensus Diagnostic Criteria for Eosinophilic Esophagitis: Proceedings of the AGREE Conference. Gastroenterology 155, 1022–1033 e10 30009819 10.1053/j.gastro.2018.07.009 PMC6174113

[86] Prahalad S (2000) Juvenile rheumatoid arthritis: linkage to HLA demonstrated by allele sharing in affected sibpairs. Arthritis Rheum 43:2335–2338 11037894 10.1002/1529-0131(200010)43:10<2335::AID-ANR22>3.0.CO;2-W

[87] Bycroft C (2018) The UK Biobank resource with deep phenotyping and genomic data. Nature 562:203–209 30305743 10.1038/s41586-018-0579-z PMC6786975

[88] Cherukuri PF (2022) Establishing analytical validity of BeadChip array genotype data by comparison to whole-genome sequence and standard benchmark datasets. BMC Med Genomics 15:56 35287663 10.1186/s12920-022-01199-8 PMC8919546

[89] Ziyatdinov A (2023) Genotyping, sequencing and analysis of 140,000 adults from Mexico City. Nature 622:784–793 37821707 10.1038/s41586-023-06595-3 PMC10600010

[90] Marees AT (2018) A tutorial on conducting genome-wide association studies: Quality control and statistical analysis. Int J Methods Psychiatr Res 27:e1608 29484742 10.1002/mpr.1608 PMC6001694

[91] Jin Y, Schaffer AA, Sherry ST, Feolo M (2017) Quickly identifying identical and closely related subjects in large databases using genotype data. PLoS ONE 12:e0179106 28609482 10.1371/journal.pone.0179106 PMC5469481

[92] Jin Y, Schaffer AA, Feolo M, Holmes JB, Kattman BL (2019) GRAF-pop: A Fast Distance-Based Method To Infer Subject Ancestry from Multiple Genotype Datasets Without Principal Components Analysis. G3 (Bethesda) 9:2447–2461 31151998 10.1534/g3.118.200925 PMC6686921

[93] Das S (2016) Next-generation genotype imputation service and methods. Nat Genet 48:1284–1287 27571263 10.1038/ng.3656 PMC5157836

[94] MacArthur J (2017) The new NHGRI-EBI Catalog of published genome-wide association studies (GWAS Catalog). Nucleic Acids Res 45:D896–D901 27899670 10.1093/nar/gkw1133 PMC5210590

[95] Watanabe K, Taskesen E, van Bochoven A, Posthuma D (2017) Functional mapping and annotation of genetic associations with FUMA. Nat Commun 8:1826 29184056 10.1038/s41467-017-01261-5 PMC5705698

[96] Consortium GT (2020) The GTEx Consortium atlas of genetic regulatory effects across human tissues. Science 369:1318–1330 32913098 10.1126/science.aaz1776 PMC7737656

[97] Kerimov N (2021) A compendium of uniformly processed human gene expression and splicing quantitative trait loci. Nat Genet 53:1290–1299 34493866 10.1038/s41588-021-00924-w PMC8423625

[98] Schmiedel BJ (2022) Single-cell eQTL analysis of activated T cell subsets reveals activation and cell type-dependent effects of disease-risk variants. Sci Immunol 7:eabm2508 35213211 10.1126/sciimmunol.abm2508 PMC9035271

[99] Andersson R (2014) An atlas of active enhancers across human cell types and tissues. Nature 507:455–461 24670763 10.1038/nature12787 PMC5215096

[100] Blanchard C (2006) Eotaxin-3 and a uniquely conserved gene-expression profile in eosinophilic esophagitis. J Clin Invest 116:536–547 16453027 10.1172/JCI26679 PMC1359059

[101] Namjou B (2014) Phenome-wide association study (PheWAS) in EMR-linked pediatric cohorts, genetically links PLCL1 to speech language development and IL5-IL13 to Eosinophilic Esophagitis. Front Genet 5:401 25477900 10.3389/fgene.2014.00401 PMC4235428

[102] Flicek P (2013) Ensembl 2013. Nucleic Acids Res 41:D48–55 23203987 10.1093/nar/gks1236 PMC3531136

[103] Trapnell C, Pachter L, Salzberg SL (2009) TopHat: discovering splice junctions with RNA-Seq. Bioinformatics 25:1105–1111 19289445 10.1093/bioinformatics/btp120 PMC2672628

[104] Love MI, Huber W, Anders S (2014) Moderated estimation of fold change and dispersion for RNA-seq data with DESeq2. Genome Biol 15:550 25516281 10.1186/s13059-014-0550-8 PMC4302049

[105] Barski A, Kartashov A (2016) BioWardrobe: an integrated platform for analysis of epigenomics and transcriptomics data. J Immunol 196 10.1186/s13059-015-0720-3 PMC4531538 26248465

[106] Ge T, Chen CY, Ni Y, Feng YA, Smoller JW (2019) Polygenic prediction via Bayesian regression and continuous shrinkage priors. Nat Commun 10:1776 30992449 10.1038/s41467-019-09718-5 PMC6467998

[107] Khera AV (2019) Whole-Genome Sequencing to Characterize Monogenic and Polygenic Contributions in Patients Hospitalized With Early-Onset Myocardial Infarction. Circulation 139:1593–1602 30586733 10.1161/CIRCULATIONAHA.118.035658 PMC6433484

